# Inhibition of Insulin Degrading Enzyme and Insulin Degradation by UV-Killed *Lactobacillus acidophilus*

**DOI:** 10.3390/medsci6020036

**Published:** 2018-05-11

**Authors:** Nadia Neyazi, Elahe Motevaseli, Mohammad Reza Khorramizadeh, Taiebeh Mohammadi Farsani, Zahra Nouri, Ensieh Nasli Esfahani, Mohammad Hossein Ghahremani

**Affiliations:** 1 Department of Medical Biotechnology, School of Advanced Technologies in Medicine, Tehran University of Medical Sciences, Tehran 1416753955, Iran; nadianeyazi@yahoo.com (N.N.); tmfarsani@gmail.com (T.M.F.); nouri1920@yahoo.com (Z.N.); 2 Department of Molecular Medicine, School of Advanced Technologies in Medicine, Tehran University of Medical Sciences, Tehran 1416753955, Iran; e_motevaseli@tums.ac.ir; 3 Biosensor Research Center, Endocrinology and Metabolism Molecular-Cellular Sciences Institute, Tehran University of Medical Sciences, Tehran 1416753955, Iran; khoramza@sina.tums.ac.ir (M.R.K.); n.nasli@yahoo.com; 4 Department of Pharmacology-Toxicology, Faculty of Pharmacy, Tehran University of Medical Sciences, Tehran 1416753955, Iran

**Keywords:** cell free supernatant, UV-killed, *Lactobacillus acidophilus* (ATCC 314), insulin degrading enzyme, insulin degradation

## Abstract

Probiotics have beneficial effects on management of type 2 diabetes (T2D). The major hallmarks of T2D are insulin deficiency and insulin resistance which emphasize insulin therapy in onset of disease. Lactobacilli such as *Lactobacillus acidophilus* (*L. acidophilus*) have well known properties on prevention of T2D and insulin resistance but not on insulin degradation. Insulin-degrading enzyme (IDE) degrades insulin in the human body. We studied the effects of cell-free supernatant (CFS) and ultraviolet (UV)-killed *L. acidophilus* (ATCC 314) on IDE activity and insulin degradation in vitro. Cell growth inhibition by CFS and UV-killed *L. acidophilus* (ATCC 314) was studied and Western blotting and a fluoregenic assay was performed to determine IDE expression and its activity, respectively. Insulin degradation was evaluated by sandwich enzyme-linked immunosorbent assay(ELISA). IDE expression and activity was reduced by CFS and UV-killed *L. acidophilus* (ATCC 314). Although, decreased enzyme expression and activity was not significant for CFS in contrast to MRL (MRS with same pH as CFS). Also, reduction in IDE activity was not statistically considerable when compared to IDE expression. Insulin degradation was increased by CFS but decreased by UV-killed *L. acidophilus* (ATCC 314).

## 1. Introduction

Probiotics are microbial functional foods which exert their beneficial effects on a host by balancing intestinal microbial properties [1], and thus influence the prevention and management of a number of clinical conditions including type 2 diabetes (T2D) [2]. Type 2 diabetes is characterized by insufficient insulin production or insulin resistance which leads to glucose metabolism disorders such as chronic hyperglycemia and dyslipidaemia [3]. As an intensive short term, insulin therapy in the early course of disease ameliorate many aspects of T2D [4].

It has been shown that probiotics increase intestinal permeability by overexpression of tight junction protein and translocation of bacterial lipopolysaccharide (LPS) and, thus, decreases metabolic endotoxemia and insulin resistance [5,6]. Two major emphasized probiotic types with lactic acid activity are *Bifidobacterium* and lactobacilli [7,8], of which the genus *Lactobacillus* is widely competitive in production of fermented food [9]. Lactobacilli are a group of Gram-positive, non-spore-forming and non-flagellated rod bacteria which are highly fermentative [10]. Their roles in lowering blood glucose levels as a consequence of insulin secretion by autonomic neurotransmission modification are well understood [11]. They also improve insulin resistance and insulin signaling pathways in the liver and maintain insulin level in plasma [12,13]. In addition to these properties, massive production, ease of administration and natural and inexpensive features have led us to consider them as appropriated alternative for insulin therapy in future [11,14]. *Lactobacillus* strains vary in their probiotic properties and potentials and *Lactobacillus acidophilus* (*L. acidophilus*) is well studied strain with well-known efficiency in this regard [1].

Despite many studies which show probiotic effects on insulin resistance, its secretion, and insulin signaling pathways, this is the first study to evaluate the effect of *L. acidophilus* on insulin degradation.

Insulin-degrading enzyme (IDE) is responsible for cellular degradation of insulin into inactive fragments [15]. Insulin-degrading enzyme is a 110-kDa zinc metalloprotease which was discovered because of the short half-life of insulin (4–6 min) in the human body. Since it has the greatest affinity for insulin, it has been considered as a potential drug target for treatment of T2D [16,17]. Some molecules and compounds such as zinc, copper, aluminum, and nitric oxide can modify the enzyme activity and thus, lead to inhibition or activation of its substrate degradation [18,19,20]. In addition, IDE gene knockout in mice has led to hyperinsulinemia and insulin resistance [21].

While probiotic interaction with host cells depends on their metabolic properties, surface molecules and secreted compounds, in this study we assumed *L. acidophilus* (ATCC 314) may alter enzyme activity and thus, decrease insulin degradation. So, the effect of cell-free supernatant (CFS) and ultraviolet (UV)-killed *L. acidophilus* (ATCC 314) on IDE activity and insulin degradation was studied on the Caco-2 cell line.

## 2. Materials and Methods

### 2.1. Preparation of Cell Free Supernatant and UV-Killed L. acidophilus

*L. acidophilus* (ATCC 314) was obtained from Pasteur Institute of Iran and cultured in MRS (de man rogosa Sharpe; Merck, Darmstadt, Germany) for 48 h in an incubator at 37 °C. Colony formation unit (CFU) was considered as 108 CFU/mL according to our previous study [22].

After this incubation time, it was centrifuged with 3800× *g* for 10 min at 4 °C. The supernatant was filtered using 0.22 µm bacterial filter and considered as cell free supernatant. Because the pH of CFS was acid (around 4), we intended MRS (with pH around 6) with decreased pH using lactic acid named as MRL (with same pH as CFS) to control acidity effects.

For preparation of UV-killed *L. acidophilus* (ATCC 314), sediment bacteria were washed twice with phosphate-buffered saline (PBS; Gibco, Darmstadt, Germany) and exposed to UV light (254 nm) for 2 h. Afterward, it was harvested again in PBS and required concentration of UV-killed bacteria was prepared before experimental usage.

### 2.2. MTT Assay

3-[4,5-dimethyl-2-thiazolyl]-2,5-diphenyl-2*H*-tetrazolium bromide (MTT) assay kit (Sigma, St. Louis, MO, USA) was used to determine the cytotoxic effects of CFS and UV-killed *L. acidophilus* (ATCC 314) on Caco-2 cells. Fifteen thousand cells were seeded in each well and incubated at 37 °C with 5% CO_2_/air atmosphere for 24 h. Then, cells were treated in triplicate with different concentration of CFS, MRS, and MRL (5%, 10%, 15%, 20%, and 25%) for 36 h and serial dilution of UV-killed bacteria (0, 250, 500, 1000, 2000, and 4000 μg/mL) for 4 h. Concentration of cells and the time of treatment were adjusted after performing different experiments. Cells with no treatment were considered as control. Cells were incubated with 100 μL MTT solution at 37 °C. Dimethyl sulfoxide (DMSO) was added after production of formazan crystals and cells were kept at dark room for 15 min. Absorbance was measured using enzyme-linked immunosorbent assay (ELISA) reader at 570 nm (Biochrom Anthons 2020, Cambridge, UK). Cell viability was determined according to below formula:

Viability (control %) = [(absorbance sample – absorbance blank)/[absorbance control – absorbance blank)] × 100

### 2.3. Cell Culture and Preparation of Cell Lysates

The Caco-2 cell line (human colonic carcinoma cell line) was purchased from Pasteur Institute, National Cell Bank of Iran and maintained in medium containing 35% Dulbecco’s Modified Eagle’s Medium (DMEM), 50% Roswell Park Memorial Institute (RPMI) 1640, 15% fetal bovine serum and 1% penicillin/streptomycin. Cells were harvested when they reached to 90% confluency and seeded again at concentration of 25 × 10^5^ cells/well. Cells kept in an incubator at 37 °C with 5% CO_2_/air atmosphere for 24 h and then treated in triplicate with 10% CFS, MRS, MRL, and 1400 µg/mL UV-killed for 2 h. Lysis buffer containing 20 mM Tris-Cl (pH 7.4), 0.5% Triton X-100, 10% sucrose was used to lyse the cells. Next cells were centrifuged in 13,500× *g* for 20 min and the supernatant was considered for experimental usage. Bicinchoninic acid (BCA) Protein Quantification Kit (pars tous biotechnology, A101251, Mashhad, Iran) was used to determine total protein concentration in samples. Cells were kept at −20 °C until the experiments were performed.

### 2.4. Western Blotting

Samples with equal concentration of total protein were separated on a 15% sodium dodecyl sulfate polyacrylamide gel electrophoresis (SDS-PAGE) gel and then transferred to Polyvinylidene fluoride or polyvinylidene difluoride (PVDF) membrane (Roche, Berlin, Germany) for 1 h at 4 °C using semi dry transfer. Unsaturated binding site was blocked by incubating filter in 1% casein blocking buffer containing Tween 0.1%. Membranes were probed with IDE rabbit polyclonal antibody (Thermo Scientific, Waltham, MA, USA) overnight at 1:2000 dilution in blocking buffer. Then membranes were washed and incubated with horseradish peroxidase-conjugated anti-rabbit IgG antibody (Bio-Rad, Hercules, CA, USA) diluted 1:6000 in 1% casein blocking buffer containing Tween 0.1%. Immunoreactive signals were measured using BM Chemiluminescence Western Blotting Kit (Roche, Berlin, Germany) and data was analyzed by Image J software (National Institutes of Health, Bethesda, MD, USA). Data was quantified by comparing the ratio of IDE signal to β-actin signal (housekeeping protein).

### 2.5. Fluoregenic Assay

Fluorogenic peptide Substrate V (7-methoxycoumarin-4-*y*l-acetyl-RPPGFSAFK-2,4-dinitrophenyl; R&D Systems, Minneapolis, MN, USA) was used to determine IDE activity in samples. Cells were incubated with pepstatin 0.1 mM, phenylmethylsulfonyl fluoride (PMSF) 0.1 mM and leupeptin 0.1 mM at 37 °C for 15 min to inhibit other peptidases and then the reaction mixture containing substrate V 10 μM, Tris 50 Mm, and NaCl 1 M, pH 7.5 was added and again incubated at 37 °C for 20 min. Fluorescence signal from the hydrolyzed substrate V was measured with excitation at 327 nm and emission at 395 nm using a Synergy 4 microplate reader (Biotek, Winooski, VT, USA). Relative IDE activity was determined by consideration of initial velocity after subtraction of background signal and intending linear regression.

### 2.6. ELISA

To evaluate insulin degradation in cell lysates human Insulin ELISA Kit (Abcam, Cambridge, MA, USA) was used based on the manufacture’s protocol. First, 300 ng/mL human recombinant insulin (Sigma, St. Louis, MO, USA) was added to cell lysates and incubated for 10 min with gentle shaking at room temperature. Then Ethylenediaminetetraacetic acid (EDTA, 1 mM) which is a metalloproteinase inhibitor was added to solution to inhibit IDE activity. Several experiments were done to adjust concentration of human recombinant insulin and time of incubation. Absorbance was measured at 450 nm and converted to ng/mL using standard curve.

### 2.7. Statistical Analysis

All tests were performed at least three times independently and the results were analyzed by one-way analysis of variance (ANOVA) followed by Tukey post-hoc test. The *p*-value less than 0.05 was considered significant.

## 3. Results

### 3.1. Cytotoxicity of Cell Free Supernatant and UV-Killed L. acidophilus (ATCC 314) on Caco2 Cell Growth

**Figure 1:**
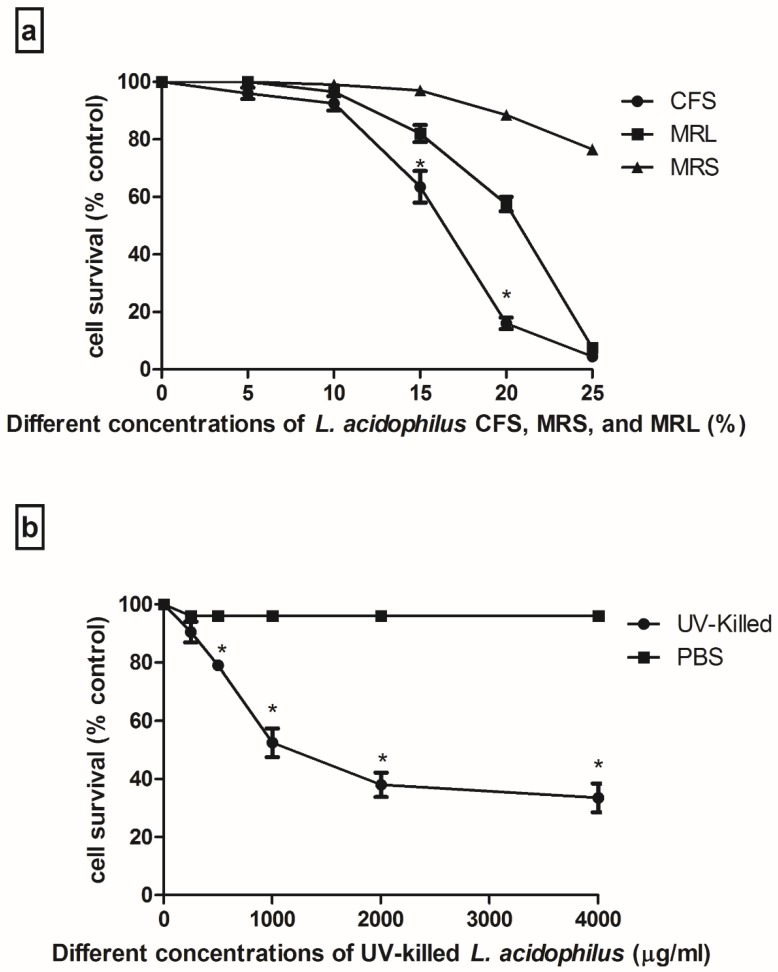
Cytotoxic effects of cell free supernatant and UV-killed *L. acidophilus* (ATCC 314) on Caco2 cell growth by 3-(4,5-dimethylthiazol-2-yl)-2,5-diphenyltetrazolium bromide (MTT) (**a**) shows treated cell with different concentration of cell free supernatant (CFS), MRS and MRL for 36 h; (**b**) represents treated cells with serial dilution of ultraviolet (UV)-killed *L. acidophilus* for 4 h. Data was shown in Mean ± standard deviation (SD). * values were significantly different from MRL, MRS; and phosphate buffer saline (PBS). (*p* < 0.05).

Figure 1 illustrates the inhibitory effects of CFS and UV-killed *L. acidophilus* (ATCC 314) on Caco-2 cell growth. It was found that CFS and UV-killed suppressed proliferation of cells in a dose-dependent manner. Although MRL caused lower cell growth, it was less cytotoxic in comparison to CFS. So, it was understood that there are some components in CFS secreted by bacteria which may lead to this outcome and acidity is not the main reason. The half maximal inhibitory concentration (IC_50_) against Caco-2 cells was 17% (*v*/*v*) for CFS and 1400 µg for UV-killed bacteria, respectively.

### 3.2. Decreased IDE Expression by Cell Free Supernatant and UV-Killed L. acidophilus (ATCC 314)

**Figure 2:**
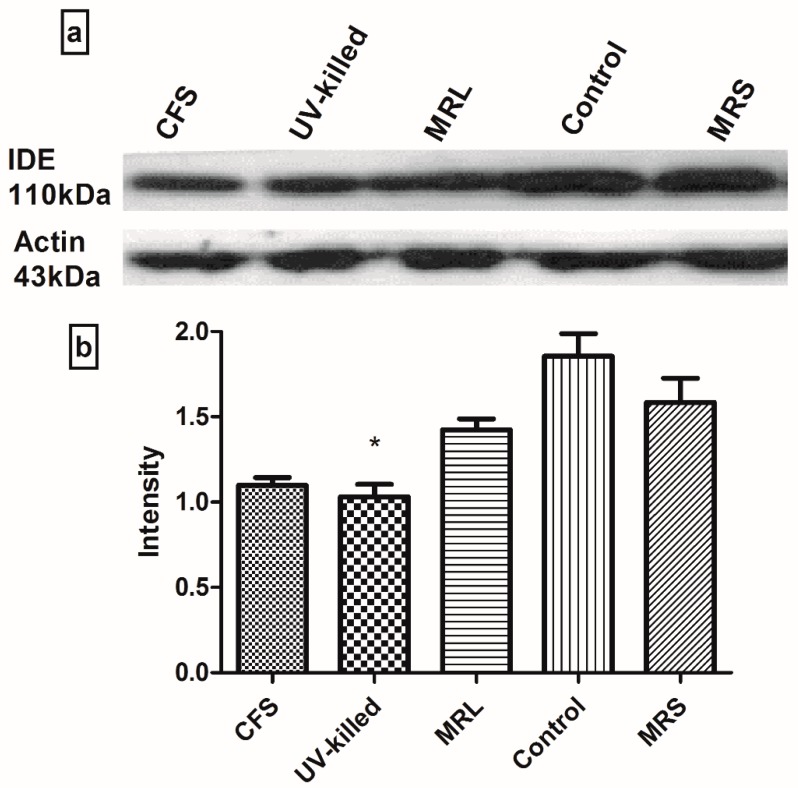
Relative IDE expression induced by CFS and UV-killed *L. acidophilus* (ATCC 314) after 2 h treatment. (**a**) Represents western blotting bands for cell lysates after 2 h treatment with 10% CFS, MRL, MRS, and 1400 µg/mL UV-killed *L. acidophilus* (ATCC 314); (**b**) represents signal intensities which were extracted using Image J. Data was analyzed by Prism (GraphPad, San Diego, CA, USA) and represented as Mean ± SD. *p < 0.05 was considered as significant.

Relative IDE expression base on Image J software analysis is represented in this study. Figure 2 shows the effects of CFS and UV-killed on IDE expression after 2 h of treatment. It was evident that CFS caused 40.77 lower expression of IDE in contrast to control, but it was not remarkable when MRL was considered. So, it was deduced that acidity may cause lower expression of IDE. However, it is shown that UV-killed significantly decrease the IDE expression by 44.4% in comparison to control.

### 3.3. Determination of IDE Activity after Treatment with Cell Free Supernatant and UV-Killed L. acidophilus (ATCC 314)

**Figure 3:**
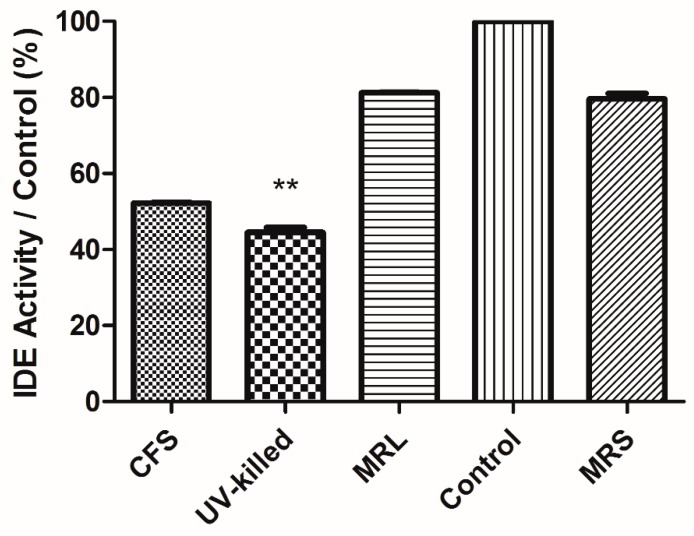
Relative IDE activity to control after treatment with CFS and UV-killed *L. acidophilus* (ATCC 314). Cell lysates were incubated with reaction mixture containing Tris 50 Mm, NaCl 1 M, pH 7.5 and fluorogenic peptide Substrate V 10 µM. Signal intensity was measured with excitation at 327 nm and emission at 395 nm. Data indicated that 47.81% and 55.49% lower IDE activity imposed by CFS and UV-killed *L. acidophilus* (ATCC 314), respectively. Reduced IDE activity induced by CFS was not significant in contrast to MRL. Data represents Mean ± SD. ** *p* < 0.01.

Enzyme activity assay showed lower activity in treated cells with both CFS and UV-killed *L. acidophilus* (ATCC 314) in contrast to controls. However, reduced IDE activity was not statistically significant for CFS in contrast to MRL. Normalized data to control (Figure 3) revealed 47.81% and 55.49% lower activity for CFS and UV-killed *L. acidophilus* (ATCC 314), respectively. We next analysed IDE activity to IDE expression which was elicited from Image J. IDE activity to IDE expression uncovered that both CFS and UV-killed decreased IDE activity in contrast to control but none of them was significant.

### 3.4. Effects of Cell Free Supernatant and UV-Killed L. acidophilus (ATCC 314) on Insulin Degradation

**Figure 4:**
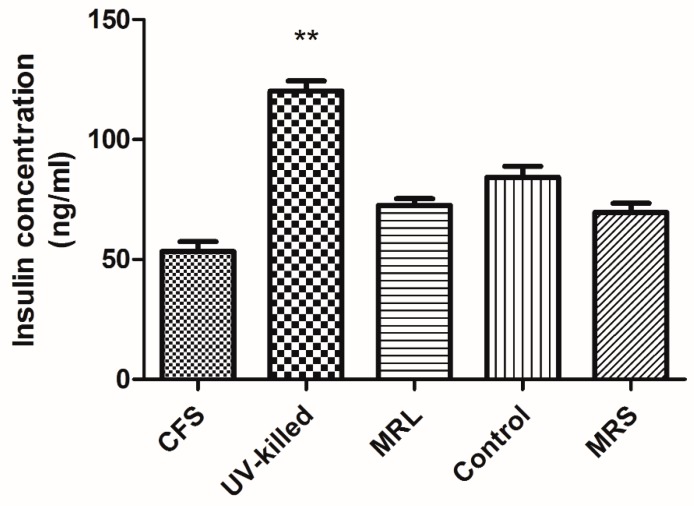
Insulin degradation by IDE using sandwich ELISA. Cell lysates were incubated with 300 ng/mL human recombinant insulin for 10 min. Ethylenediaminetetraacetic acid EDTA (1 mM) was added to the solution for the inhibition of IDE activity. It was found that CFS decreased the insulin level but it was not considerable in comparison to MRL. UV-killed *L. acidophilus* (ATCC 314) significantly reduced insulin degradation in contrast to control. Result are shown as mean ± SD and *p* < 0.05 was considered significant (** *p* < 0.01).

Figure 4 demonstrates insulin degradation after 10 min incubation of treated and control cells with human recombinant insulin. It is figured out that CFS imposes 36.15% higher insulin breakdown in contrast to control but this was not significant compared to MRL. In contrast, UV-killed *L. acidophilus* led to 42.85% lower insulin degradation which is significant compared to control.

## 4. Discussion

Recent genomic, metagenomic and metabolomic studies have shown the efficacy of probiotics, especially lactobacilli and bifidobacteria as prospective biotherapeutics in the prevention and treatment of T2D [23].

The dominant hallmarks of T2D are progressive pancreatic β cell degeneration and insulin resistance coupled with higher insulin secretion from pancreatic β cell and lower insulin degradation [24]. Insulin-degrading enzyme mainly controls insulin levels either by uncoupling insulin from insulin receptor (IR) or by removal and degradation of insulin partially or completely. Therefore, inhibition of IDE may increase insulin senility and IR activity [25,26].

It has been observed that *L. acidophilus* adheres to the Caco-2 cell line and exerts its effects either by cell-to-cell interaction or by secreted compounds [27,28]. In addition, it was previously indicated that the Caco-2 cell line has high amounts of IDE [29].

In this study, we evaluated the effects of CFS and UV-killed *L. acidophilus* (ATCC 314) on IDE activity and insulin degradation in Caco-2 cells.

MTT assay showed suppressed cell proliferation after treatment with CFS for 2 h. MRS with same pH as CFS MRL led to lower cell growth but it was less cytotoxic in comparison to CFS which leads us to understand some other secreted substrates by bacteria may cause cell growth inhibition. This is consistent with a previous study done by Soltan Dallal and colleagues who indicated that CFS of *L. acidophilus* significantly inhibits Caco-2 cell growth [30]. Ultraviolet-killed *L. acidophilus* (ATCC 314) also had cytotoxic effects on Caco-2 cells in a dose-dependent manner. It is likely that bacteria have similar effects on Caco-2 cells [31,32]. In addition, heat-killed *L. acidophilus* has inhibitory effects on cell growth by their soluble polysaccharides [33]. This is in compliance with our study which indicated that lactobacilli supernatant suppress cell viability by downregulating caspase-3 mRNA levels [34].

The gene encoding IDE protein is located on 10q23–q24 [35]. This enzyme is greatly released in cytosol but it is also found in peroxisomes and in secreted form [36]. Insulin-degrading enzyme expression and action leads either to increase insulin degradation to inactive form and thus, reduce insulinemia or to decrease insulin senility by interrupting the IR signaling pathway [37]. We found 40.77% and 44.4% lower IDE expression for CFS and UV-killed *L. acidophilus* (ATCC 314). The reduced expression was not significant for CFS in contrast to MRL and it was shown that acidity may cause this outcome. However, it was shown that UV-killed *L. acidophilus* (ATCC 314) significantly decreased the IDE expression compared to control. Similar to our study, it was recently found that nutrition restricted diet imposes lower IDE expression and thus, lower clearance in protein-restricted diet mice [38]. Moreover, it was found that cafeteria diet has decreased protein and mRNA levels of IDE in skeletal muscle and liver of mice and reduces insulin clearance [37]. Another study has indicated that free fatty acids, glucagon and pioglitazone increase IDE activity in diet-induced obese mice [17].

Several mechanisms are effective in enzymatic activity of IDE, including the ability to create chemical changes in the cysteines present in IDE, using enzyme inhibitor or a positive modulator of IDE. Researchers suggest that sometimes compounds bind to cysteines of the enzyme in T2D and change them, thus, inhibiting IDE activity irreversibly [39]. These compounds can also alter the equilibrium state of the enzyme between dimer and tetramer and thus, increase or decrease IDE activity [40]. Enzyme activity assays showed 47.81% and 55.49% lower activity for CFS and UV-killed *L. acidophilus* (ATCC 314), respectively. However, none of them was significant when IDE activity was compared to IDE expression. Since optimal pH for enzymatic activity is 6.0–8.5, it is logical that CFS causes lower IDE activity in contrast to control which indicated here [15]. In this regard, past studies have pointed to free long chain fatty acids and ubiquitin as causes of lower IDE activity [41,42].

Insulin-degrading enzyme mediates insulin degradation in the human body and produces inactive fragments of A and B chains [16]. Our study indicates that CFS increased insulin breakdown in contrast to control but it was not significant compared to MRL. UV-killed decreased insulin degradation which is statistically considerable compare to control. Earlier, it was shown that *L. acidophilus* preserved insulin sensitivity after 4 weeks of supplementation by volunteers [12]. Also, *L. acidophilus* and *Bifidobacterium* in combination or alone significantly increased insulin secretion in serum [39].

## 5. Conclusions

In conclusion, CFS and UV-killed *L. acidophilus* (ATCC 314) led to a decrease in IDE expression and its activity. Insulin-degrading enzyme expression and its activity were not considerable when CFS was compared to MRL and it is deduced that acidity may cause this result. Reduction in IDE activity was not significant when data compared to IDE expression was considered. CFS increased insulin degradation while UV- killed *L. acidophilus* (ATCC 314) significantly decreased insulin degradation. Our study suggests the need of further knowledge to explore the effects of bacterial cell wall on insulin degradation and sensitivity to evoke possible molecules and signaling pathways. Also, future research may be carried out to find novel targets in the management of T2D.
